# Modulating Hyperpolarization-Activated Cation Currents through Small Molecule Perturbations: Magnitude and Gating Control

**DOI:** 10.3390/biomedicines11082177

**Published:** 2023-08-02

**Authors:** Cheng-Shih Chen, Edmund Cheung So, Sheng-Nan Wu

**Affiliations:** 1 Department of Anesthesia, An Nan Hospital, China Medical University, Tainan 70965, Taiwan; chengshih.chen@gmail.com (C.-S.C.); edmundsotw@gmail.com (E.C.S.); 2 School of Medicine, National Sun Yat Sen University College of Medicine, Kaohsiung 804, Taiwan; 3 Department of Medical Education & Research, An Nan Hospital, China Medical University, Tainan 70965, Taiwan; 4 Department of Physiology, National Cheng Kung University Medical College, Tainan 701, Taiwan

**Keywords:** hyperpolarization-activated cation current, current kinetics, small molecules, herbal drugs

## Abstract

The hyperpolarization-activated cation current (*I*_h_) exhibits a slowly activating time course of the current (*I*_h_) when the cell membrane is hyperpolarized for an extended duration. It is involved in generating electrical activity in various excitable cells. Numerous structurally distinct compounds or herbal drugs have the potential to impact both the magnitude and gating kinetics of this current. Brivaracetam, a chemical analog of levetiracetam known to be a ligand for synaptic vesicle protein 2A, could directly suppress the *I*_h_ magnitude. Carisbamate, an anticonvulsant agent, not only inhibited the *I*_h_ amplitude but also reduced the strength of voltage-dependent hysteresis (Hys_(V)_) associated with *I*_h_. Cilobradine, similar to ivabradine, inhibited the amplitude of *I*_h_; however, it also suppressed the amplitude of delayed-rectifier K^+^ currents. Dexmedetomidine, an agonist of α2-adrenergic receptor, exerted a depressant action on *I*_h_ in a concentration-dependent fashion. Suppression of *I*_h_ amplitude was observed when GAL-021, a breathing control modulator, was present at a concentration exceeding 30 μM. Lutein, one of the few xanthophyll carotenoids, was able to suppress the *I*_h_ amplitude as well as to depress Hys_(V)_’s strength of *I*_h_. Pirfenidone, a pyridine derivative known to be an anti-fibrotic agent, depressed the *I*_h_ magnitude in a concentration- and voltage-dependent fashion. Tramadol, a synthetic centrally active analgesic, was shown to reduce the *I*_h_ magnitude, independent of its interaction with opioid receptors. Various herbal drugs, including ent-kaurane-type diterpenoids from Croton tonkinensis, Ganoderma triterpenoids, honokiol, and pterostilbene, demonstrated efficacy in reducing the magnitude of *I*_h_. Conversely, oxaliplatin, a platinum-based chemotherapeutic compound, was observed to effectively increase the *I*_h_ amplitude. Collectively, the regulatory effects of these compounds or herbal drugs on cellular function can be partly attributed to their perturbations on *I*_h_.

## 1. Introduction

**Figure 1:**
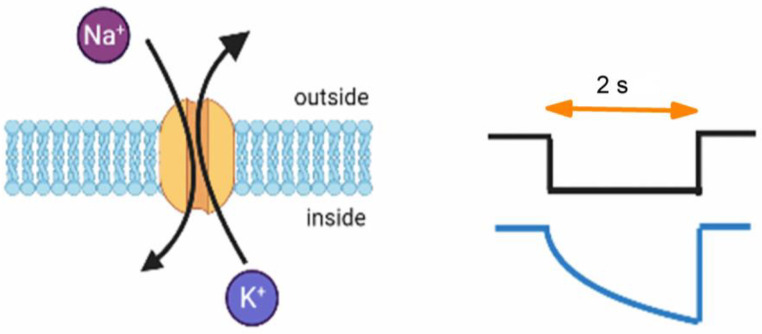
Simplified representation graph of a hyperpolarization-activated cyclic nucleotide-gated (HCN) channel. The image on the right displays a voltage-clamp protocol. The top graph illustrates the voltage command applied for hyperpolarization, while the bottom graph represents the waveform of the hyperpolarization-activated cation current (*I*_h_). This demonstrates the characteristic slow activation of *I*_h_ without an accompanying inactivation process during prolonged membrane hyperpolarization. The image on the left depicts the conductance of the HCN channel in its open state. This ion channel is known to exhibit permeability comparable to that of both Na^+^ and K^+^ ions. In this state, Na^+^ ions flow from the extracellular space into the intracellular space, while K^+^ ions move in the opposite direction, both driven by the electrochemical gradient.

The hyperpolarization-activated cation current, also known as the *I*_h_ or funny current (*I*_f_), plays a crucial role in generating repetitive electrical activity in various types of cells, such as heart cells, neurons, and neuroendocrine or endocrine cells [1,2,3,4,5,6,7,8,9,10]. This specific type of ionic current involves a combined flow of Na^+^ and K^+^ ions (Figure 1), demonstrating distinctive ion selectivity. These currents exhibit an inwardly rectifying property whereby their amplitude and activation increase in response to more hyperpolarizing potentials [1,2,4,11,12]. The current can be inhibited by CsCl or ivabradine. Given its tonic activity in resting cells, the activation of this current occurs at the resting membrane potential and primarily results in an inward current carried by Na^+^ ions. Based on this observation, it can be inferred that some cells do not exhibit a truly resting membrane potential and thus deviate from the traditional notion of cellular resting states [8]. The presence or assumption of *I*_h_, as demonstrated in respective models, is crucial because the inward current it generates induces membrane depolarization, playing a pivotal role in initiating action potentials in excitable cells [1,4,8,11]. Recent studies from a computational model of layer V pyramidal cells have described that the slow kinetics of *I*_h_ in response to a long hyperpolarizing step can produce long-lasting, activity-dependent modification of membrane excitability in different excitable cell types [13].

**Figure 2:**
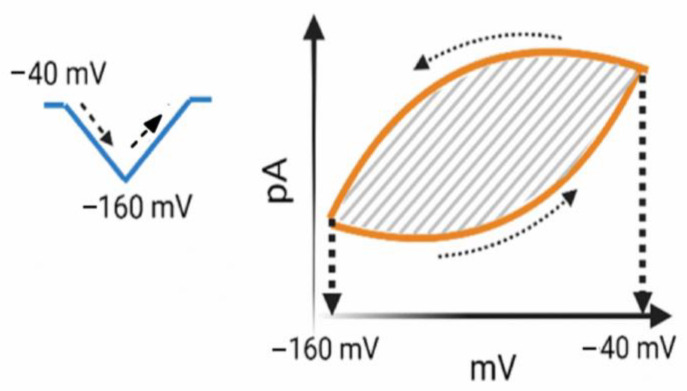
Simplified representation graph of the voltage-dependent hysteresis (Hys_(V)_) of *I*_h_. On the left, a representation diagram of a long-lasting inverted triangle represents a ramp voltage (Vramp, indicated in blue). The dashed arrow indicates the change in voltage over time. On the right is a graph representing the relationship of voltage versus whole-cell *I*_h_ current, specifically illustrating the voltage-dependent hysteresis (Hys_(V)_) (depicted in orange color). The bold dotted lines are positioned at membrane potentials of −40 and −160 mV, respectively. The light dotted curve arrows adjacent to the orange line indicate the counterclockwise direction of the current flow over time. The gray shaded area in the diagram represents the hysteresis strength and is enclosed by the *I*_h_ current during both the descending (forward) and ascending (backward) limbs of the triangular V_ramp_. The *I*_h_ current induced by the descending limb of the triangular V_ramp_ is noticeably smaller than the current induced by the ascending limb of the V_ramp_.

Upon long-lasting inverted triangular ramp voltage (V_ramp_), the forward and backward amplitudes of *I*_h_ were noted to be distinct, reflecting the presence of non-equilibrium voltage-dependent hysteresis (Hys_(V)_) of *I*_h_ (Figure 2) [14,15,16,17]. The Hys_(V)_ property of *I*_h_ is considered to serve a role in affecting the overall behaviors of excitable cells, including pituitary GH3 cells. In other words, a shift of ion-channel mode in which the voltage sensitivity in gating the charge movement of the current is dependent on the previous state of the HCN channel involved [14,16]. When the membrane potential of an excitable cell undergoes repolarization or hyperpolarization, specifically during the descending limb of the isosceles-triangular V_ramp_, the current strength of *I*_h_ is relatively small according to the Hys_(V)_ loop (Figure 2). However, during the depolarization of the cell membrane, correspondingly, in the ascending limb of this triangular V_ramp_, the magnitude of *I*_h_ significantly increases, leading to a substantial alteration in the membrane potential. As a result, the depolarization of the cell membrane becomes notably pronounced due to the influence of *I*_h_ current.

These ionic currents are attributed to channels known as hyperpolarization-activated cyclic nucleotide-gated (HCN) channels, as demonstrated previously [6,11,18,19]. Several mammalian subtypes, namely HCN1, HCN2, HCN3, and HCN4, have been cloned, as reported previously [11,12,18,19]. These subtypes can combine to form either homo- or heterotetramers, each exhibiting distinct biophysical properties. The activation kinetics of HCN1 channels typically activate more slowly compared to HCN2 channels. HCN1 channels have more negative voltage dependence, meaning that they activate at more hyperpolarized potentials compared to HCN2 channels [11,12]. However, it is important to note that these are general trends, and specific kinetic properties can vary depending on the experimental conditions and cellular context. Functional expression of HCN2, HCN3, or a combination of HCN2 and HCN3 channels has been observed in pituitary GH3 cells or other types of endocrine cells [5,6,7,8]. Considering the significance of *I*_h_ (i.e., currents mediated by HCNx) in contributing to the excitability and automaticity of excitable cells [12,13,20], any compounds capable of influencing the magnitude, gating behaviors, or voltage-dependent hysteresis (Hys_(V)_) of *I*_h_ can exert a substantial impact on the functional activities of these excitable cells.

In this review paper, our aim was to present information on recently identified compounds or herbal drugs (refer to Table 1) that have a notable influence on the intensity, gating kinetics, and Hys_(V)_ behavior of the current (known as *I*_h_). These compounds have a distinct and significant effect on *I*_h_, many of which differ from their originally developed targets. The IC_50_ or EC_50_ values needed for the regulation of *I*_h_ magnitude are illustrated in Table 2. Therefore, their impact on *I*_h_ should be considered an additional and important mechanism. It is necessary to further clarify the influence of these compounds on cellular function. These effects could offer new insights and enhance our understanding of the potential use of the HCN channel in combating specific types of diseases [11,21].

## 2. Compounds That Are Known to Inhibit *I*_h_

### 2.1. Brivaracetam

Brivaracetam (Brivact^®^, Brivlera^®^, (2S)-2-[(4R)-2-oxo-4-propylpyrrolidin-1-yl]butanamide), a chemical analog of levetiracetam, is an orally or intravenously bioavailable racetam derivative with anticonvulsant properties [36,37]. Brivaracetam has also been reported to attenuate pain behavior in a murine model of neuropathic pain [36,38]. Recent investigations have shown that, in addition to the inhibition of voltage-gated Na^+^ currents, brivaracetam at a concentration greater than 10 μM was also found to suppress the amplitude of *I*_h_ in pituitary GH_3_ cells [22]. GH_3_ cells are not neurons; they are a cell line derived from rat pituitary tumor. Moreover, according to a simulated firing of action potentials generated from the modeled neuron, the firing frequency and amplitude of action potentials were found to be reduced in the presence of brivaracetam [22]. Therefore, it is possible that besides being a high affinity ligand for synaptic vesicle protein 2A (SV2A) [39], brivaracetam can directly perturb the ionic currents, including *I*_h_, hence showing a potential additional impact on the functional activities of different excitable cells.

### 2.2. Carisbamate

Carisbamate (RWJ-333369, (RS)-2-(2,3-dihydro-1,4-benzodioxin-6-yl)ethyl carbamate), a bioactive orally administered neuromodulator, has been shown to be beneficial for the treatment of different types of convulsive disorders, including drug-resistant focal epilepsy and partial onset seizure [40,41,42]. Previous studies have reported that carisbamate prevents the development and production of epilepsy-like discharges and exerts neuroprotective effects after epilepticus-like injury [40,43]. Of interest, a recent study reported that carisbamate caused a concentration-dependent decrease in *I*_h_ amplitude, with an IC_50_ value of 38 μM [24]. There was also a marked retardation of the activation time course of *I*_h_ in response to a 2-s hyperpolarizing command voltage. The presence of carisbamate also suppressed the Hys_(V)_ strength of *I*_h_ activation in response to a long-lasting isosceles-triangular Vramp, suggesting that this drug may interact with the voltage-sensing domains of the HCN channel. Apart from its ability to inhibit voltage-gated Na^+^ current, carisbamate-mediated changes in the magnitude, gating kinetics, and Hys_(V)_ behavior of *I*_h_ may also be of pharmacological or therapeutic relevance [24]. Moreover, the *I*_h_ has been shown to be functionally present in heart cells [1,3,10]. Therefore, the carisbamate-mediated inhibition of *I*_h_ seen in excitable cells may be responsible for its ability to attenuate the increase of heart rate induced by exposure to organophosphate administration, as described previously [43,44]. Further research is also needed to explore the extent to which carisbamate’s inhibition of *I*_h_ contributes to its anticonvulsant effects.

### 2.3. Cilobradine

Cilobradine (DK-AH269, 2-[(3-bromo-5-isoproxy-2-methylphenyl)methylamino]-N-(2,3-dimethylphenyl)acetamide) has been shown to suppress the activity of HCN channels in mouse sinoatrial node cells [45]. Previous observations have revealed its effectiveness in modifying *I*_h_ in pancreatic α- or β-cells, thereby influencing hormone secretion [7,46]. In a recent study [26], the effective IC_50_ required for the cilobradine-induced inhibition of *I*_h_ was estimated to be 3.38 μM, a value that tends to be higher than that for its suppression of HCN channels identified in mouse sinoatrial cells. The presence of cilobradine was also noted to alter the impedance amplitude profile of *I*_h_ in response to chirp voltage [26]. Furthermore, cilobradine was able to suppress delayed-rectifier K^+^ currents (*I*_K(DR)_) along with an increase in the inactivation time course of the current. As the cilobradine concentration increased from 1 to 3 μM, the midpoint of the steady-state inactivation curve of *I*_K(DR)_ was shifted along the voltage axis towards hyperpolarizing voltage by approximately 7 mV with no change in the gating charge of the curve during exposure to 1 or 3 μM cilobradine [30]. It is also important to note that exposure to cilobradine has been previously reported to modulate balance function, given that it may concertedly influence functional HCN channels in vestibular hair cells of the inner ear [47] and the K_V_3.1 channels, which are enriched in the auditory pathway [48]. In other words, the presence of cilobradine may synergistically act on *I*_h_ and *I*_K(DR)_ to influence the functional activities of excitable cells.

### 2.4. Dexmedetomidine

Dexmedetomidine (Precedex^®^, (S)-4-[1-(2,3-dimethylphenyl)ethyl]-1H-imidazole), a lipophilic imidazole derivative, is viewed as a potent and selective agonist of the α2-adrenergic receptor [49,50,51]. Previous studies have revealed that this drug exerts a variety of actions on the human brain, such as sedation, anesthetic-sparing effects, and analgesia [52,53,54]. However, there is evidence to highlight the notion that direct interactions with membrane ionic channels may be an unidentified but important mechanism underlying dexmedetomidine-induced action in central neurons [28,53,55,56,57]. In particular, in pituitary GH_3_ cells, dexmedetomidine produced a depressant action on *I*_h_ in a concentration- and time-dependent fashion, with an IC_50_ or K_D_ value of 1.21 or 1.97 μM, respectively [28]. Cell exposure to dexmedetomidine shifted the steady-state activation curve of *I*_h_ toward a more hyperpolarized potential. This drug also diminished the Hys_(V)_ strength of *I*_h_ during a long-lasting triangular V_ramp_. In pheochromocytoma PC12 cells, the presence of dexmedetomidine also suppressed *I*_h_ effectively [28].

It is important to highlight the time-dependent effect of dexmedetomidine on *I*_h_. When cells were exposed to dexmedetomidine, it was observed that the time course of *I*_h_ activation during prolonged hyperpolarizing pulses slowed down. These findings suggest that the molecule has a higher affinity for the open state of HCN channels, specifically during sustained hyperpolarization, than for closed or resting channels in GH_3_ cells. This implies that, besides its known ability to bind to α2-adrenergic receptors, dexmedetomidine may directly influence the activation process of the HCN channel, thereby modifying the magnitude and kinetics of *I*_h_ in response to prolonged membrane hyperpolarization. Additionally, the blockade of *I*_h_ induced by dexmedetomidine could serve as a significant ionic mechanism that effectively reduces the intrinsic membrane excitability of neurons, as well as neuroendocrine or endocrine cells, in vivo [7,28,52,53,54,55,58].

However, it needs to be noted that the sedative properties of dexmedetomidine and its effects on the thalamocortical network might not be primarily influenced by the direct inhibition of *I*_h_. This implies that dexmedetomidine likely acts through other mechanisms, such as α_2_-adrenoceptor activation and modulation of noradrenergic excitation, to exert its sedative effects [53].

### 2.5. GAL-021tion

GAL-021 (N2-methoxy-N2-methyl-N4,N6-dipropyl-1,3,5-triazine-2,4,6-triamine or N-[4,6-bis-n-propylamino-(1,3,5)-triazin-2-yl]-N,O-dimethylhydroxyamine) has been developed as a novel breathing control modulator thought to preserve respiratory drive and to protect patients from the respiratory impairment resulting from opioids and other modalities [59]. Previous studies have reported that this agent is an experimental drug demonstrated to inhibit Ca^2+^-activated K^+^ channels with large conductance functionally expressed on type 1 cells of the carotid bodies [59,60].

Consistent with previous reports [60], recent findings have shown the ability of GAL-021 to suppress depolarization-evoked Ca^2+^-activated K^+^ currents in GH_3_ cells [30]. However, GAL-021 at a concentration greater than 30 μM was found to inhibit the amplitude of *I*_h_ elicited by long-lasting membrane hyperpolarization [30]. If both Ca^2+^-activated K^+^ current and *I*_h_ are inhibited simultaneously, their individual inhibitory and excitatory effects on neuronal excitability are counteracted. The inhibition of Ca^2+^-activated K^+^ currents reduce the hyperpolarizing influence, while the inhibition of *I*_h_ reduces the depolarizing influence. It has also been reported that active respiratory neurons express functional HCN channels [61]. Consequently, in addition to the known inhibition of Ca^2+^-activated K^+^ currents [30], the actions of GAL-021 on excitable cells may partly result from the suppression of *I*_h_ amplitude.

### 2.6. Lutein

Lutein (3,3′-dihydroxy-α-carotene-6,6′-diene), a xanthophyll carotenoid known as β,ε-carotene-3,3′-diol, is derived from a hydride of a (6R)-β,ε-carotene. It is found in various vegetables and fruits, but notably, it is present in high concentrations in the macula of the human retina, where it acts as a yellow filter [62]. It is a pigment that belongs to the carotenoid family, and its yellow color allows it to selectively absorb certain wavelengths of light. Specifically, lutein absorbs blue and ultraviolet light while allowing other wavelengths, including yellow and longer visible light, to pass through. Recent studies provide evidence that the dietary intake of lutein can lead to the accumulation of lutein in retinal neural tissue, thereby potentially promoting eye and brain health [62,63]. Of interest, a recent report showed that as pituitary GH_3_ lactotrophs were exposed to lutein, the magnitude of *I*_h_ can be inhibited in a concentration-, state-, voltage-, and Hys_(V)_-dependent manner [32]. The IC_50_ value required for the inhibition of *I*_h_ was 4.1 μM [32]. These results reflect that, besides its antioxidative or anti-inflammatory properties, the presence of lutein can inhibit the magnitude of *I*_h_ as well as alter gating and Hys_(V)_ behavior. The lutein’s action would engage in the modifications of spontaneous action potentials present in excitable cells (e.g., GH_3_ cells), presuming that similar in vivo observations occur.

The functional expression of HCN2, HCN3, or a combination of HCN2 and HCN3 channels, has been reported in GH_3_ cells [6,8]. Therefore, it seems unlikely that the lutein-induced inhibition of *I*_h_ in native cells is specific to a particular isoform. However, there is a possibility that lutein’s blockage of HCN channels may be related to alterations in phosphene perception in the retina [64]. Why lutein in physiological concentrations induces phosphene perception remains to be further studied. However, the observed effect of lutein on specific ionic currents has the potential to contribute to the beneficial effects of lutein in retinal conditions, particularly macular degeneration [65,66]. It would be worthwhile to further investigate whether lutein exhibits selectivity towards different HCN channel isoforms.

### 2.7. Pirfenidone

Pirfenidone (Esbriet^®^, 5-methyl-1-phenyl-2(1H)-pyridinone), a pyridine derivative, is thought to act by interfering with the production of transforming growth factor-β and tumor necrosis factor-α [67]. Mounting evidence has shown the effectiveness of pirfenidone either in treating idiopathic pulmonary fibrosis, or in non-small cell lung cancer [67,68]. A previous report showed that the presence of pirfenidone can inhibit the amplitude of *I*_h_ in a concentration- and voltage-dependent fashion [34]. Additionally, when GH_3_ cells were exposed to pirfenidone, the activation time course of *I*_h_ became slower in response to sustained membrane hyperpolarization. These findings suggest that the blocking effect of pirfenidone on *I*_h_ is not immediate but develops with time after the HCN channel opens, leading to a significant delay in current activation. In addition, exposure to pirfenidone resulted in the suppression of the Hys_(V)_ strength of *I*_h_, which was elicited by a long-lasting triangular V_ramp_. This suggests that pirfenidone or compounds with similar structures could bind to the open state of the channel and/or inhibit prolonged channel opening [34]. Therefore, this study provides evidence that pirfenidone has the potential to modify specific ionic currents. Such modifications could have implications for therapeutic applications, particularly when pirfenidone is applied to different excitable cells. These findings suggest that pirfenidone might yield additional beneficial effects in various contexts.

### 2.8. Tramadol

Tramadol ((±)-cis-2-[(dimethylamino)methyl]-1-(3-methoxyphenyl)cyclohexanol) is a synthetic centrally active analgesic, and its clinical use is rapidly increasing. The mechanism of its analgesic actions was thought to feature mixed μ-opioid and non-opioid activity [69]. Of particular interest, increasing evidence has emerged that this drug may be a direct modulator of ion channels that include HCN channels [23]. The presence of tramadol produced a block of *I*_h_ in a time- and concentration-dependent manner. This drug at a concentration of 10 μM could shift the activation curve of *I*_h_ to more negative potentials, with no change in the slope’s steepness of the curve. Tramadol reduced the firing of spontaneous action potentials in GH_3_ cells, indicating the tonic activity of *I*_h_ in non-voltage-clamped cells [23]. Thus, the direct blockade of *I*_h_ by tramadol may partially contribute to the rhythmic activity of neurons or neuroendocrine cells, and similar results are observed in vivo [11,70].

## 3. Herbal Drugs That Are Known to Inhibit *I*_h_

### 3.1. Ent-Kaurane-Type Diterpenoids (i.e., Croton-01, Croton-02, Croton-03) from Croton Tonkinensis

The genus croton (*Euphorbiaceae*) includes about 300 species that are distributed throughout tropical regions. *C. tonkinensis* Gagnep is a tropic shrub native to northern Vietnam and has been used to exert anti-inflammatory and cancer chemopreventive activities [71]. Earlier reports have shown that the compounds purified from croton could modify different types of ion channels [25,72]. For example, the presence of croton-03 (ent-1β-acetoxy-7α,14β-dihydroxykaur-16-en-15-one) has been shown to suppress the *I*_h_ amplitude in pituitary GH_3_ cells and INS1 insulin-secreting cells [25]. The hysteretic strength of *I*_h_ elicited by triangular Vramp was effectively attenuated by adding croton-03. In current-clamp potential recordings, the amplitude of the sag potential in response to long-lasing hyperpolarizing stimuli was also suppressed by the croton-03 presence [25]. Croton-03 also shifted the activation curve of *I*_h_ to a more hyperpolarized potential, with no change in the gating charge of the curve. The sag potential is associated with the activation of HCN channels. These channels generate a specific current called the *I*_h_. The sag potential and the underlying *I*_h_ current play important roles in regulating membrane excitability and rhythmic activities [25]. Regarding the steady-state activation curve of *I*_h_ during exposure to croton-03, the voltage for half-maximal activation was found to be in the range of the firing of action potentials. Furthermore, the presence of croton-03 resulted in a slower activation time course of *I*_h_ at different voltage levels [9]. This suggests that the croton-03 molecule has a higher affinity for the open state of HCN channels compared to the closed or resting state of the channels found in GH_3_ or INS-1 cells. As a result, the degree of *I*_h_ blockage caused by croton-03 seems to vary depending on the applied voltage. Moreover, any alterations in *I*_h_ amplitude and gating due to croton-03 would be influenced by factors such as the concentration of croton-03, the occurrence of action potentials, and the preexisting resting potential.

### 3.2. Ganoderma Triterpenoids (Active Constituents of Ganoderma Spores)

Ganoderma mushrooms (Lingzhi in Chinese, or Reishi in Japanese) are a traditional Chinese herbal medicine that has been used as a nutritional supplement [73]. The triterpenoid fraction of Ganoderma, consisting of more than 300 lanostane-tetracyclic compounds, has been shown to be effective at exerting various biological actions, such as that known either to provide antioxidant activities or to produce neuroprotective effects [74,75]. Recent work has demonstrated the ability of *Ganoderma triterpenoids* to suppress the magnitude and alter the gating kinetics of *I*_h_. The results reflect that these triterpenoids can modify a dose-, time-, and state-dependent activation of *I*_h_ in GH_3_ cells and in HL-1 cardiomyocytes [27]. The IC_50_ value required for the *Ganoderma triterpenoids*-mediated block of *I*_h_ was estimated to be 11.7 μg/mL. These triterpenoids were reported to contain various nucleosides, including adenosine [27,75]. However, the triterpenoid-mediated inhibition was not reversed by further addition of adenosine receptor antagonists [27]. The current-clamp voltage recordings were also found to decrease the firing of spontaneous action potentials and the magnitude of sag potentials in GH3 cells [27]. It also appears unlikely that the triterpenoid-induced inhibition of *I*_h_ in GH3 cells results from nucleosides (e.g., adenosine) possibly contained in their ingredients. The inhibitory action on *I*_h_ caused by *Ganoderma triterpenoids* may thus have a profound impact on the electrical behaviors of excitable cells (e.g., endocrine, and heart cells) if similar in vitro or in vivo findings occur.

### 3.3. Honokiol

Honokiol (3′,5-di-(2-propenyl)-1,1′-biphenyl-2,2′-diol) is a hydroxylated biphenyl compound obtained from *Magnolia officinalis* and from other species of the family *Magnoliaceae*, and has been used in traditional Asian medicines (Hou p’u or Saiboku-tu(o)) [76]. Honokiol is recognized as a potential natural compound that has been shown to exert multiple effects on various cellular responses in different cancer models [77]. Previous investigations have also shown the ability of magnolia bark or honokiol to modify the secretion of catecholamines from the adrenal medulla [78]. At the cellular level, honokiol, or magnolol has been shown to induce Ca^2+^ mobilization in cortical neurons and neuroblastoma cells [79]. Recent work has also shown that honokiol or other similar structural compounds can interact with the HCN channels to alter the magnitude and gating of *I*_h_ during the long-lasting hyperpolarization step in excitable cells, namely GH_3_ cells and Rolf B1.T olfactory neurons [29]. The steady-state activation curve of *I*_h_ in GH_3_ cells shifted toward a negative voltage in the presence of honokiol. However, the lack of an effect on the gating charge of the curve occurred, reflecting that the honokiol action on the channel might act as a gate to open the channel but not, instead, act on the region that senses the transmembrane potential. Honokiol also suppress Hys_(V)_’s strength of *I*_h_ elicited during triangular V_ramp_. This compound was also found to suppress the firing of spontaneous action currents measured under cell-attached current recordings in GH_3_ cells, and this action is thought to be mediated largely by inhibitory action on *I*_h_ [29]. Therefore, the inhibition of *I*_h_ was noted to be rapid in onset and is therefore likely to be responsible for its modulatory action on the functional activities of sensory neurons or endocrine cells.

### 3.4. Pterostilbene

Pterostilbene (3′,5′-dimethoxy-4-stilbenol) is a natural demethylated analog of resveratrol and was named after a natural phenolic compound found in *Pterocarpus marsupium Roxb (Fabaceae*), which is native to India, Nepal, and Sri Lanka. It is one of the active compounds in the extracts of *P. marsupium* that was used in Ayurvedic medicine for the treatment of various cancers (Ahmad and Rajagopal, 2015). The evidence has shown its inhibitory effects on almost every cellular event that promotes tumor progression toward metastasis in an apoptosis-dependent or apoptosis-independent manner [80,81,82]. Recent evidence has shown that the application of pterostilbene to GH_3_ cells resulted in the inhibition of *I*_h_ in a concentration-dependent manner with an IC_50_ of 0.84 μM [31]. The presence of pterostilbene increased the activation time constant of *I*_h_ elicited by long-lasting membrane hyperpolarization. During exposure to 1 μM pterostilbene, the steady-state activation curve of *I*_h_ was distinctly shifted to more hyperpolarizing potentials by about 11 mV, producing channel opening at more negative voltages. However, like resveratrol [83], pterostilbene can stimulate the magnitude of Ca^2+^-activated K^+^ currents in pituitary GH_3_ cells and in hippocampal mHippoE-14 neurons [31]. Therefore, its modifications on ion-channel activity could conceivably be one of the ionic mechanisms underlying pterostilbene-mediated actions, if similar in vitro or in vivo results can emerge in neurons, and in neuroendocrine or endocrine cells.

## 4. The Compound That Is Known to Stimulate *I*_h_

### Oxaliplatin

Oxaliplatin (Eloxatin^®^, cis-[oxalato(1R,2R-diaminocyclohexane)platinum(II)]) belongs to a family of platinum-based chemotherapeutic compounds. In combination with 5-fluorouracil, this drug has been used in the treatment of advanced colorectal or gastric cancers [84,85]. Despite its good safety profile, its use has been found to confer susceptibility to peripheral neuropathy, affecting sensory and motor nerve fibers, explaining the unsuitability for long-term treatment [21,86]. Recent investigations have revealed the ability of oxaliplatin to modify the magnitude of membrane ionic currents, including *I*_h_ [21,34,35]. A previous report showed that the presence of oxaliplatin can exert dual stimulatory actions on two types of ionic currents, namely *I*_h_ and membrane electroporation-induced current (IMEP). Unlike those of *I*_h_, the biophysical properties of macroscopic IMEP are virtually stochastic and not yet deterministic. Oxaliplatin-induced stimulation of *I*_h_ could be found in pituitary GH_3_ and R1220 cells and in Roif B1.T olfactory sensory neurons [33]. Rat pituitary R1220 cells were supplied by ScienCell Research Laboratories (Carlsband, CA, USA), https://sciencellonline.com/rat-pituitary-cells, accessed on 1 March 2023.

It is important to note that the oxaliplatin concentration used to block *I*_h_ is closely similar to that achieved in the plasma of treated patients (i.e., 3.6–5.6 μM) [87]. The stimulation by oxaliplatin of *I*_h_ observed in GH_3_ cells was not instantaneous and occurred in a time- and concentration-dependent fashion. Moreover, the effects of oxaliplatin on membrane ionic currents were noted to be rapid in onset [33], and they can thus be upstream of the formation of platinum-DNA adducts occurring inside the nucleus [88]. Moreover, the inhibition of *I*_h_ caused by exposure to either dexmedetomidine, lutein, pirfenidone, or ent-kaurane-type diterpenoids from *C. tonkinensis* can be effectively counteracted by the subsequent addition of oxaliplatin [25,26,89,90].

## 5. Conclusions

**Figure 3:**
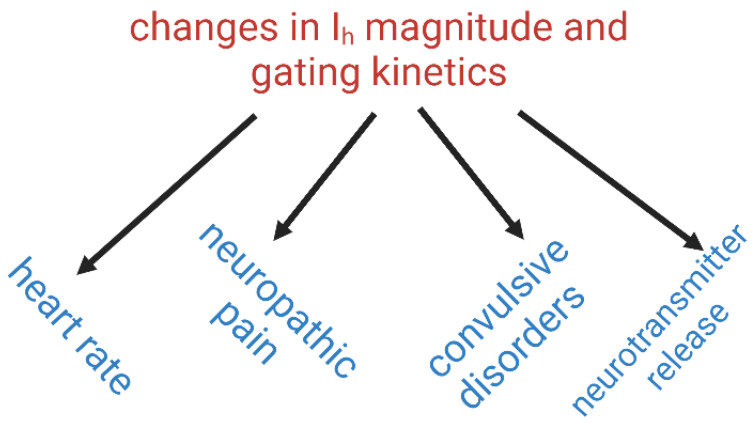
Changes that occur when the magnitude and gating properties of *I*_h_ are modified. Each solid arrow represents a specific type of channel that may occur.

As described above, and in published studies, experimental observations have also revealed that a variety of compounds or herbal drugs may directly modulate the magnitude of *I*_h_. Table 3 shows the two-dimensional chemical structures of the compounds presented herein. The modifications of *I*_h_ induced by these compounds can impact heart rate, improve neuropathic pain, and demonstrate anti-convulsant effects (Figure 3). Furthermore, owing to the slow activation properties of *I*_h_, which lacks an inactivation process, the time constant for current activation can occasionally extend to around one second, suggesting that this time is closely related to the synaptic delay. The synaptic delay, which refers to the brief period of time it takes for an electrical signal to travel across a synapse, usually ranges from about 0.3 to 5 ms depending on the specific synapse. Several important HCN modulating compounds, including endogenous cytokines, have also been reported, including EC18 [91], and different derivatives of ivabradine like zatebradine/cilobradine [45] or clonidine [92]. EC18 is an important lead structure due to its moderate selectivity. Furthermore, HCN is influenced by endogenous cytokines, such as interferons and interleukins, which could contribute to indirect modulation pathways of the HCN channels [93]. Therefore, when the cells or tissues studied have functional expression of HCNx channels, modifying the magnitude and/or gating properties of *I*_h_ will affect the release of neurotransmitters from the presynaptic neuron, thereby influencing synaptic transmission (Figure 3) [11,19,21,70]. Furthermore, the development of compounds that are isoform-specific for HCNx channels would result in modality-specific treatments. Since many herbal drugs do indeed have significant effects on ion channels, attempting to extract and purify these herbal drugs will also be an important issue in future drug development.

## Figures and Tables

**Table 1:** Compounds presented in this paper that can inhibit the hyperpolarization-activated cation current (*I*_h_), as well as compounds that can stimulate this current.

Compounds known to inhibit *I*_h_
a. Brivaracetam b. carisbamate c. cilobradine d. dexmedetomidine e. GAL-021 f. lutein g. pirfenidone h. tramadol
2. Herbal drugs known to inhibit *I*_h_
a. *ent*-krauane-type diterpenoids b. *Ganoderma* triterpenoids c. honokiol d. pterostilbene
3. Compound known to stimulate *I*_h_
a. Oxaliplatin

**Table 2:** Summary showing IC_50_ or EC_50_ values for modulating *I*_h_ amplitude as indicated in the paper.

Compound	IC_50_	Refs.	Compound	IC_50_	Refs.
Brivaracetam	greater than 25 μM	[22]	Tramadol	13.6 μM	[23]
Carisbamate	38 μM	[24]	Croton-01 *, croton-02 *, and croton-03 *	2.89, 6.25, and 2.84 μM	[25]
Cilobradine	3.38 μM	[26]	Ganoderma triterpenoids	11.7 μg/ml	[27]
Dexmedetomidine	1.21 μM	[28]	Honokiol	2.1 μM	[29]
GAL-021	greater than 30 μM	[30]	Pterostilbene	0.84 μM	[31]
Lutein	4.1 μM	[32]	Oxaliplatin	1.3 μM **	[33]
Pirfenidone	3.65 μM	[34]			

* belongs to *ent*-kaurane-type diterpenoids. ** The value represents the EC_50_, since oxaliplatin was found to activate the *I*_h_ amplitude [35].

**Table 3:** Two-dimensional chemical structures of the compounds presented in this paper.

Compound	Chemical Structure
Brivaracetam	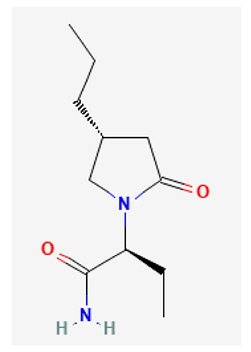
Carisbamate	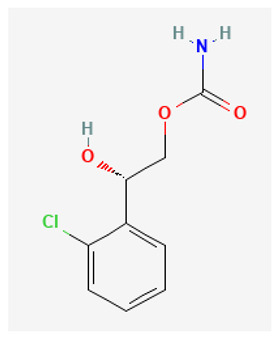
Cilobradine	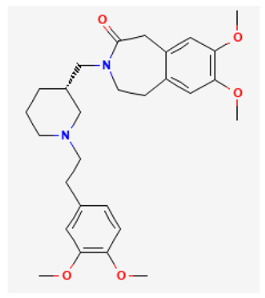
Dexmedetomidine	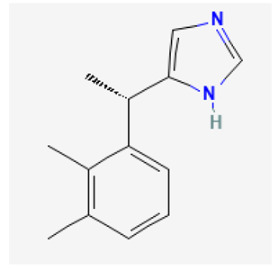
GAL-021	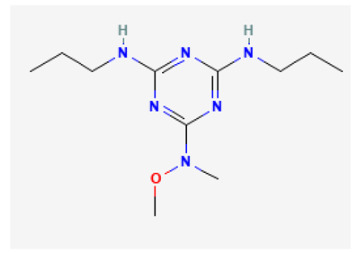
Lutein	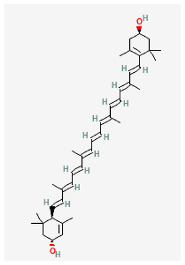
Pirfenidone	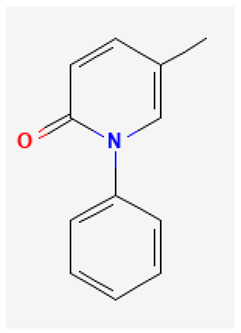
Tramadol	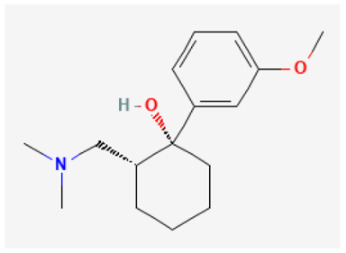
Croton-01, croton-02, and croton-03	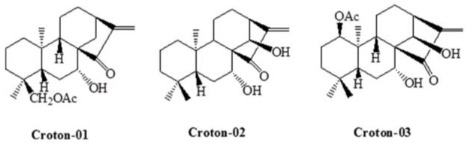
Honokiol	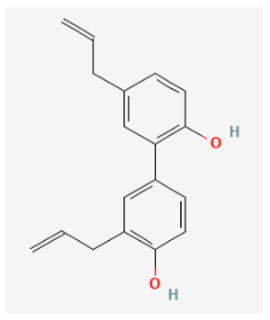
Pterostilbene	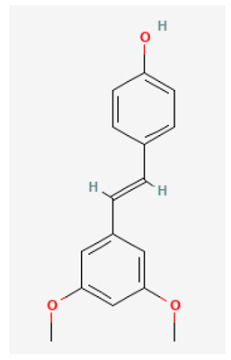
Oxaliplatin	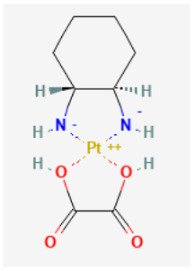

## Data Availability

The original data are available upon reasonable request to the corresponding author.

## Abbreviations

HCN channel	hyperpolarization-activated cyclic nucleotide-gated channel
Hys_(V)_	voltage-dependent hysteresis
V_ramp_	ramp voltage
*I*_h_**	hyperpolarization-activated cation current
